# Optimizing Participant Engagement in Cyberhealth Co-Design: Course-of-Action Framework Analysis

**DOI:** 10.2196/70772

**Published:** 2025-08-27

**Authors:** Melanie Tremblay, Christine Hamel, Anabelle Viau-Guay, Dominique Giroux

**Affiliations:** 1 École Nationale d'Administration Publique, 555, boulevard Charest Est, Québec, QC, G1K 9E5, Canada, 1 4186413000; 2 Department of Teaching and Learning Studies, Centre de recherche et d'intervention sur la réussite scolaire, Laval University, Québec, QC, Canada, Québec, QC, Canada; 3 Department of Rehabilitation, Université Laval, Québec, QC, Canada; 4 Center of Excellence on Aging Quebec, Québec, QC, Canada; 5 Centre intégré universitaire de santé et de services sociaux de la Capitale-Nationale, Québec, QC, Canada

**Keywords:** engagement, co-design, caregivers, activity analysis, course-of-action framework, affordances

## Abstract

**Background:**

Co-design is recognized for its potential to enhance the usability of products through active user participation. However, participation alone does not guarantee the effectiveness of the resulting product. Understanding participants’ engagement during co-design activities can provide valuable insights into their motivations, concerns, and contributions, which are critical to achieving successful outcomes.

**Objective:**

This study aims to analyze participant engagement in a digital health co-design parent project focusing on developing a tool to facilitate support-seeking for elderly caregivers.

**Methods:**

The parent project included 74 participants from 3 categories: caregivers, health care and social service professionals, and community workers. Testimonies for this study were collected from 20 participants using the self-confrontation interview methodology. Engagement was analyzed qualitatively using the course-of-action framework. The engagements were organized into emergent themes. The analysis focused on variations in engagement patterns across participant categories and sessions.

**Results:**

A total of 3 themes of engagement were identified: tool design, participant needs, and contextual situations. Engagement was distributed similarly across themes, except for community workers, who were more focused on needs (52/94, 42%) than tool design (25/62, 20%). There was significant variation in engagement over sessions, with tool design being more prominent during specific sessions (co-design sessions CoD5, CoD7, and CoD8) and less important during others (CoD4, AC2 [advisory committee session], CoD6, and AC3). Activities directly tied to design tasks significantly enhanced engagement with tool design. These results underscore the influence of activity types in shaping participants’ focus and involvement.

**Conclusions:**

This study highlights the role of affordances in co-design activities to balance engagement across design, collaboration, and participation dimensions. By strategically leveraging affordances, future co-design projects can optimize engagement and ensure more effective outcomes.

## Introduction

### Background

Digital health can improve access to and quality of health care, lower costs, and provide a personalized experience for patients, but it also raises ethical challenges [1], usability problems [2], and, as seen, few successful implementations [3]. Involving end users in the creation of digital health solutions can minimize usability and accessibility problems [4]. Although co-design has several theoretical virtues, it does not guarantee positive outcomes. A total of 2 systematic literature reviews identified mixed and even negative outcomes, including communication problems, misunderstandings between users and the development team leading to conflicts, higher user expectations, and a negative influence on data quality [5-7]. What factors lead to better results?

For example, there is a great diversity of activities conducted during co-design sessions: interviews, advisory council meetings, focus groups, surveys, deliberative methods [8], and plenary sessions [9]. Gray literature also offers a wide range of techniques for collaborative sessions, problem-solving, and ideation that can be used in co-design [10-12]. How does the type of activity affect outcomes?

Extra time is needed when involving patients in health research [13]. To date, research on co-design in digital health is still seldom described, evaluated, or empirically tested, raising questions about the cost-effectiveness of user contributions compared to the investments required in time, effort, and money [8]. This paper contributes to the body of work on co-design initiatives with a focus on what drives participants—namely, their engagement—in a co-design project, based on a strong theoretical lens on human activity and empirical results provided by the participants themselves.

### Positioning Engagement and Co-Design Through a Theoretical Lens

Co-design in this study aligns with the perspective of Sanders and Stappers [14], which involves users (or potential users) in the development of knowledge, concepts, and idea generation. For us, co-design lies at the intersection of 3 dimensions: design, collaboration, and participation. First, participants engage in “design as a reflective practice,” following Schön [15], to reflect on their situation and contemplate potential solutions to improve it. The second dimension is collaboration, where actors engage in mutual learning [16-20], leveraging each other’s knowledge and experience to better understand the situation and co-create the solution. The third dimension is participation, which involves how actors act, speak, and think [21], and how they may ultimately benefit from their participation beyond the co-design project’s objectives [22]. We perceive participation and collaboration as interrelated dimensions rather than levels on a ladder [23].

Engagements of participants can help understand participation in co-design by providing insights into what motivates or concerns a person to participate (act, speak, or think; see Textbox 1 ). For example, an actor might have concerns about the capability of users with low digital literacy. These engagements affect their participation and their willingness to engage in design as a reflective practice and in mutual learning about the situation. They can influence the potential for collaboration, creativity, and ultimately the innovative, useful, and usable potential of the proposed solution.

Textbox 1. **Plain language summary.**

- Affordances: perceptual cues in the environment that suggest specific interactions.
- Course-of-action framework: theoretical and analytical framework for activity analysis that focuses on understanding an individual’s lived experience by examining their actions in context, including their intentions, concerns, perceptions, and adaptations over time.
- Engagement: what motivates or concerns a person to participate (act, speak, and think).
- Pragmatism paradigm: a paradigm that is not committed to any sort of philosophical stance and oriented toward what works.
- Self-confrontation interviewing: one-on-one discussion in which participants are shown excerpts of recordings from a recent activity to stimulate reflection and verbalization about their actions and experiences.

Participant engagements in co-design initiatives, particularly in the digital health domain, are crucial but come with inherent challenges. These challenges include the multifaceted nature of digital health implementations, the lack of empirical evaluations [8], and the need for a precise understanding of engagement based on participants’ perspectives and theoretical frameworks.

### Previous Work

The concept of engagement is often blurred with involvement [8,24-26] or remains unclear [7,27,28]. In both cases, engagement seems to be defined by how actors were involved in the projects. A total of 3 studies provide concise definitions, positioning engagement as involvement: “actively collaborate” [29]; “partnership, collaboration or consultation” [13]; and engagement type: co-production, co-design, and co-creation [30]. These are extrinsic considerations, not necessarily focusing on the motivations or concerns from the actors’ perspectives.

Jolliff et al [31] used an existing framework proposed by the Patient-Centered Outcomes Research Institute [32], including extrinsic but also intrinsic considerations, such as engagement quality (perceptions of actors) and partners’ outcomes (effects for actors). Peters et al [33] also associated quality of engagement with emotional and cognitive factors.

Some studies report on engagement without referencing co-design or participative design, instead focusing on patient partners [27] or patient and community involvement in research [7,13,29]. While actors were involved in these projects, they were not engaged in the co-creation of the solution, which does not align with our perspective on co-design. Empirical studies focusing on engagement [7,25,27], along with literature reviews on engagement [8,13,26,29], provide insights into factors affecting engagement and offer recommendations based on the results.

Among the problems affecting engagement, power distribution or tokenism (symbolic participation) was reported in all papers except in O’Connor et al [26], whose literature review included only 4 papers discussing co-design. Papers also mentioned:

- Levels of expertise, lack of skills, or limited knowledge [7,13]
- Unclear contribution to the project [13]
- Expectation of the degree and level of involvement not clearly articulated, leading to a lack of motivation [7]
- Communication issues [7,13]
- Personal life, values, and the engagement and recruitment approach [26]
- Limited availability of actors, lack of awareness and understanding of guiding frameworks and validated methods, and resource constraints (time and money) for adequate planning [29]

Papers mentioned positive emotional outcomes for actors (targeted end users), such as increased confidence in their daily lives [8], a sense of pride and accomplishment, feeling empowered, an increased knowledge and skills [8,13]. Most papers conclude with recommendations for future research, including:

- Clear communication [7,8,27]
- Clear end-user expectations and clearly defined roles for all parties involved in co-design [8,29]
- Engaging actors as early as possible and continuing engagement throughout the process [7,28-31,33]
- Allocating sufficient time to achieve objectives [8,27]
- Fostering a positive environment and mutual respect [29]
- Providing orientation, support, education, and training to help actors participate [8,27,29,31]
- Authentic design participation focused on “designing with” rather than “designing for” [25]
- Evaluation of engagement [29,31,33]

There was still a lack of empirical results or insufficient reporting of methodology across all the papers. Most papers did not provide detailed information about the co-design activities, except for reviews of wireframe workshops [7] and a design charrette [25]. Slattery et al [8] noted that the most frequent activities in their review were interviews or advisory councils, which do not explain how actors contributed to the co-creation process.

### Objective of This Study

Despite the critical role of participant engagement in co-design for achieving fruitful outcomes, discussions on engagement remain predominantly focused on extrinsic factors, blurred with involvement, and are poorly empirically described. While the insights are useful for optimizing the co-design process, connecting them to participants’ motivations or concerns from their perspective remains challenging.

In this study, engagement is considered differently, based on what participants found significant from their own perspective, aligned with the course-of-action framework [21]. This article aims to address this gap by providing an intrinsic description of participant engagements in a co-design situation.

## Methods

### Study Design

#### Overview

We used a mixed-methods approach [34], within the pragmatism paradigm [35]. In our study, the pragmatism paradigm provided various perspectives on participants’ engagements, each building upon the other [36]. Most of the methodology was guided by the course-of-action framework [21].

#### Engagement in the Course-of-Action Framework

The course-of-action provides a strong theoretical and analytical framework for documenting participant experiences in digital health co-design initiatives [37]. Within the course-of-action framework, engagement represents the actor’s significant concerns or motivations related to the elements considered in the situation [38]. Engagements are shaped by participants’ histories and what is meaningful or significant to them at that moment [39]. Engagement is also directly linked to expectations, as expectations modulate engagement. Concerns and motivations lead actors to expect that something will happen in relation to them. Engagement is also influenced by the referential (knowledge and experience) of the actors. In a co-design situation, we examine how the participants’ engagements evolve, what motivates them to think, speak, or act (participate). Expectations related to engagements can hinder the actor’s participation if they are engaged by other elements in the situation, diverting them from the task [40,41].

### Study Setting: The Co-Design Parent Project

This engagement study is embedded in a larger multisite co-design parent project (from May 2017 to June 2018), whose objective was to develop a tool to facilitate the search of resources for caregivers of seniors experiencing loss of autonomy. A complete description of the co-design study protocol is available in Latulippe et al [42], and the results of the approach are presented in 3 articles [43-45].

**Figure 1.**
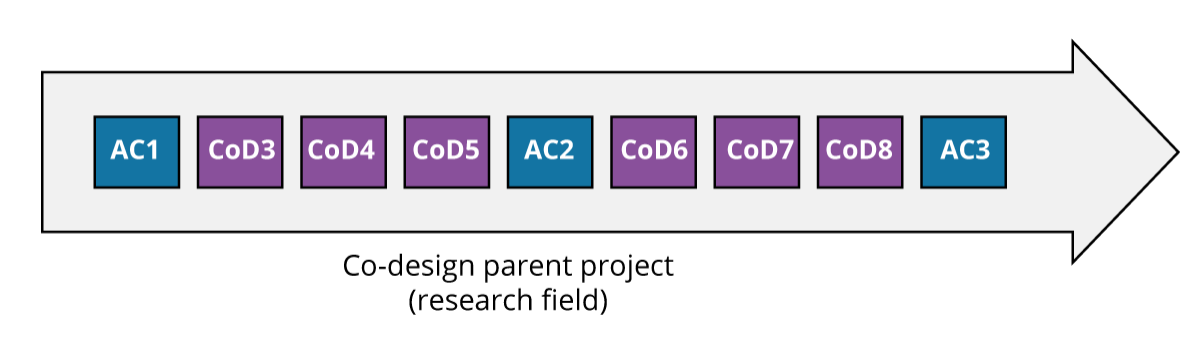
Co-design sessions (CoD) and advisory committee sessions (AC) of the parent project.

Data collection was conducted as part of a co-design project aimed at designing a tool to facilitate resource searches for caregivers of seniors experiencing loss of autonomy. A total of 8 co-design sessions (CoDs) were held from May 2017 to June 2018. These sessions were interspersed with 3 advisory committee sessions (ACs) (see Figure 1).

The following describes the CoD workshops and the AC meetings:

- Regional CoD workshops (n=8): Conducted in 11 administrative regions of Québec, each workshop gathered local caregivers, health and social service professionals (HSSPs), and community-organization staff to surface region-specific needs and generate design ideas. As attendance was location-based, membership naturally differed from one CoD workshop to the next.
- AC meetings (n=3): Convened in Québec City, the AC also gathered local caregivers, HSSPs, and the community-organization staff. Its mandate was to ensure alignment between the progress of the tool’s design and the data collected during the co-design sessions, resolve outstanding design questions, and steer iterative prototype development. The participants in the AC sessions were generally the same individuals, as all these sessions took place in the Quebec region.

The co-design process culminated in the creation of a prototype website with 2 main sections: one for caregivers to search for resources and another for service providers to offer resources. The prototype was the subject of a study based on the triangulation of 3 frameworks in usability and user experience [46].

### Ethical Considerations

The parent co-design project received institutional review board approval before the first workshop began (Université Laval #2017‐103, issued April 20, 2017). The initial certificate covered the core workshop activities and brief contextual questionnaires completed by HSSPs and community-organization staff; these questionnaires informed the broader design effort but are not analyzed in this engagement study.

An amendment adding video-recorded participatory observation and postsession self-confrontation interviews was submitted in June 2017 and was approved on July 17, 2017, after CoD1 (May 2017) and CoD2 (early July 2017) had already taken place. Consequently, no postsession interviews were conducted for those 2 early workshops; full data collection commenced with AC1 and CoD3 and continued through AC3 and CoD8. All participants involved in the amended protocol provided written informed consent specific to these additional procedures and received a nominal compensation of CAD $20 (US $16.45). Data were anonymized for this study.

### Recruitment for This Study

Participants for this study were recruited during the sessions. A caregiver serving on the AC was interviewed twice—immediately after AC1 and again after AC3—to capture how his engagement trajectory evolved over the life of the project. Retaining both interviews enabled a longitudinal within-person analysis consistent with the course-of-action framework; discarding the second interview would have forfeited this analytic leverage.

### Data Collection

This co-design project served as the research setting for our study, which aimed to describe the engagements of participants in a cyberhealth co-design project. Data collection occurred in 5 steps (see Textbox 2).

Textbox 2. **The 5 steps of the data collection.**

Ethnographic data collection: internet-based questionnaires for HSSPs and community organizations, along with semistructured interviews with caregivers. Conducted under the parent codesign project; not analyzed in this study.

- Participatory observation of the sessions: video and audio recordings (3 h) of the sessions to capture data on participants’ activities during the session.
- Partial chronicles (extrinsic description): the day following the session, a synthesized transcription of the complete session was made using the recordings to reconstruct the sequence of events with the help of time markers. Significant moments for participants (interpreted by the researcher) were selected.
- Self-confrontation interviews: interviews of approximately 1 hour conducted mostly within 2 days after the activity. Participants were presented with portions of recordings representing key moments of the session, aiming to obtain verbalizations about their activities during the session. The partial chronicle was used to structure the interview with the temporal sequence of the session, allowing the researcher to question the participant and identify significant moments expressed by the participant. Video and audio recordings were made during the interviews.
- Complete chronicles: transcriptions of the self-confrontation interviews were used to complement the partial chronicle of the session with participants’ verbalizations (intrinsic description).

### Data Analysis

The analysis was conducted in 2 stages by the primary author. The first stage (deductive) involved reconstructing the participants’ course-of-action in the complete chronicle. Engagements and expectations were mostly identified by paraphrasing participants’ statements to clarify the researchers’ understanding, while in some cases, verbatim excerpts were used to stay as close as possible to participants’ experiences.

These documents were then used in the second stage (inductive), conducted using the qualitative and mixed methods analysis software MAXQDA 2020 (VERBI Software). Engagements were organized into emergent themes [47], describing the nature of the engagements.

We subsequently cross-referenced the themes with variables, including participant categories (caregivers, HSSPs, and organizations) and sessions. This step allowed us to generate tables presenting the frequency with each theme. This quantitative analysis was useful for selecting data based on what appeared significant to a larger number of participants, from their perspective, and for maintaining analytic validity by guarding against potential biases when selecting data to present [47].

## Results

### Participants

The co-design project included 74 participants. We collected testimonies from 20 participants, including caregivers (n=9), HSSPs (n=4), and community workers (n=7; see Table 1). We conducted 21 interviews (1 AC participant was interviewed twice).

**Table 1.** Session’s location and participants.

Session	Location	Participants
AC^a^1	Centre-du-Québec	Caregiver (n=1) HSSP^b^ (n=1)
CoD^c^3	Saguenay	Community worker (n=1)
CoD4	Bas-Saint-Laurent	HSSP (n=1) Community worker (n=1)
CoD5	Outaouais	Caregiver (n=2) Community worker (n=2) HSSP (n=1)
AC2	Centre-du-Québec	Community worker (n=1) Caregiver (n=1)
CoD6	Montréal-Laval	HSSP (n=1) Caregiver (n=1)
CoD7	Mauricie	Caregiver (n=2)
CoD8	Montérégie	Caregiver (n=1) Community worker (n=1)
AC3	Centre-du-Québec	Community worker (n=2)

a AC: advisory committee meeting.

b HSSP: health and social service professionals.

c CoD: co-design session.

The AC sessions were conducted entirely in plenary, where participants were invited to express their positions on specific issues where consensus had not been reached during the CoD sessions. All CoD sessions began with a plenary presentation outlining the project, the objectives of the session, and other relevant information. Subsequently, participants were divided into small workshops of 3 to 4 people, each facilitated by a member of the research team and typically focused on a different subcomponent or angle of the session’s main objective, allowing for complementary contributions to the overall session goal.

A distinctive feature of CoD5 was that all small groups worked collaboratively on the same specific task: creating a paper prototype. In contrast, other sessions (CoD1 to CoD4 and CoD6 to CoD8) explored the session’s objective through more diverse or exploratory angles within subgroups. Participants had the freedom to choose their workshops, resulting in subgroups that were sometimes homogeneous and sometimes heterogeneous in terms of participant categories. Activities varied based on the session objectives, the progress of the tool design, and the specific goals of the subgroup workshops. The analysis revealed 3 main themes of participants’ engagements: engagements related to their needs (personal and professional), engagements related to the situation, and engagements related to the design of the tool.

### Nature of Engagements Per Participant Category

**Figure 2.**
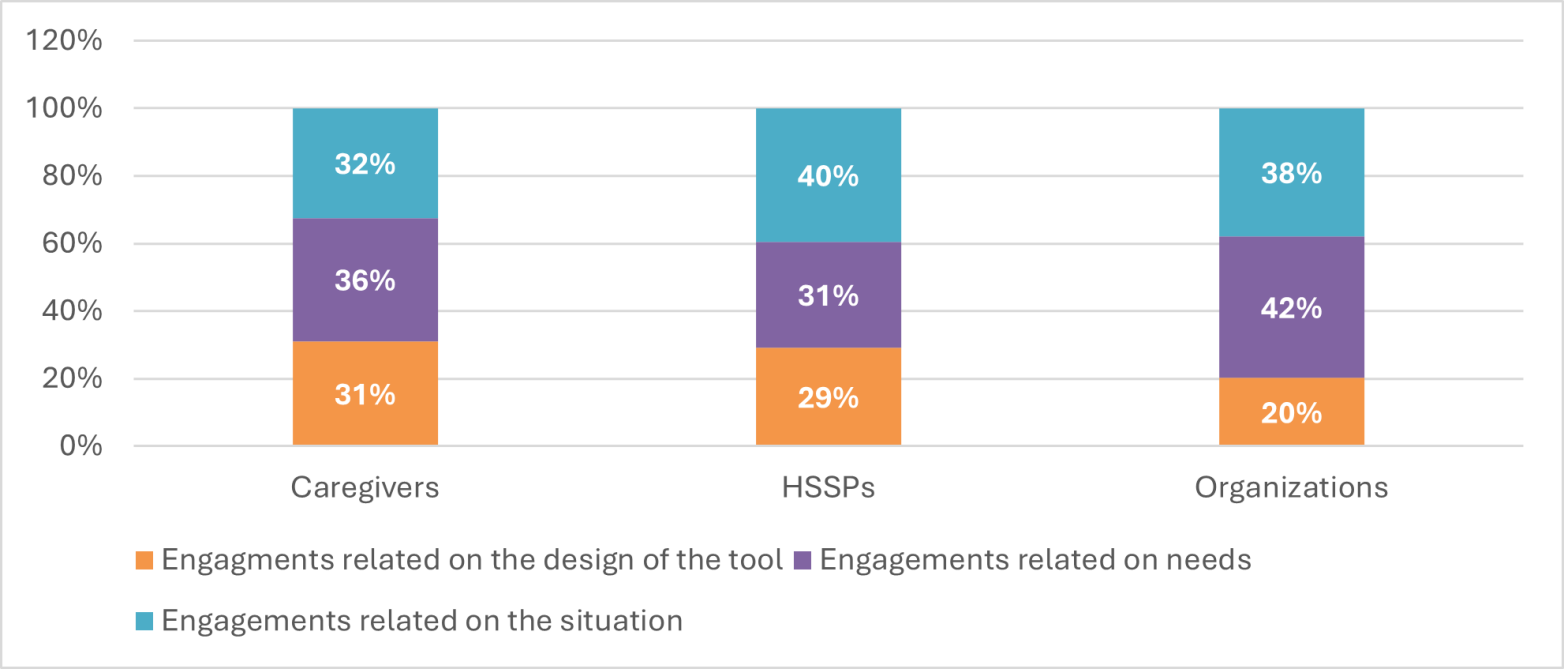
Distribution of engagement by participant category. HSSP: health and social services professional.

The distribution of engagement was quite similar among caregivers and HSSPs across themes (see Figure 2). However, the engagement of organizations (community workers) was significantly less focused on tool design.

### Nature of Engagements Per Session

**Figure 3.**
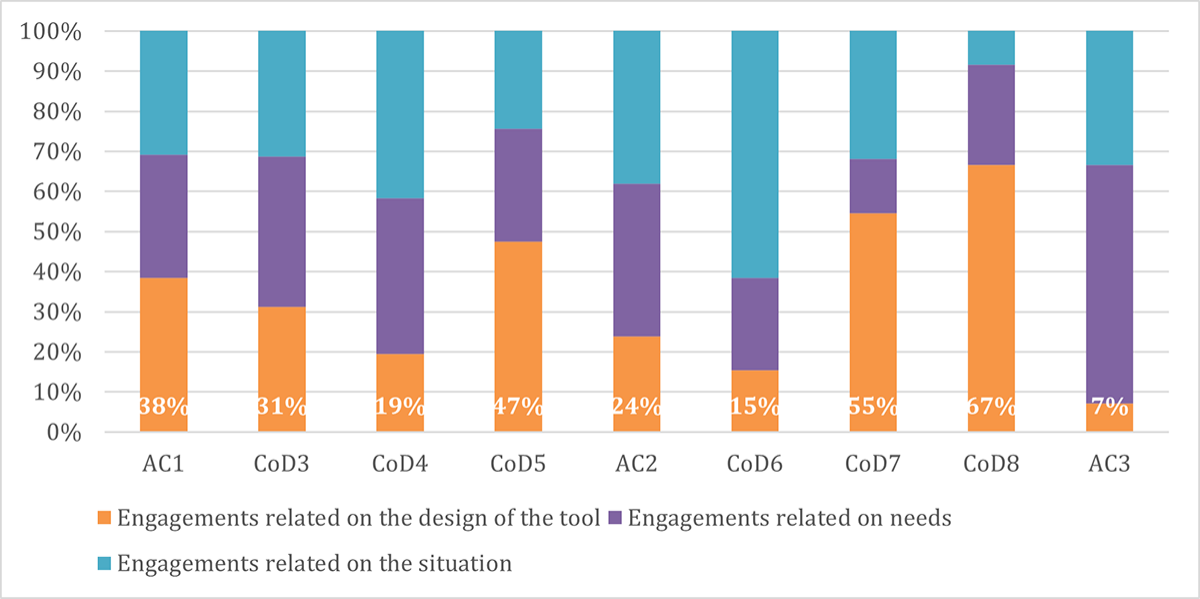
Evolution of the nature of engagement by session. AC: advisory committee meeting; CoD: co-design session.

Figure 3 shows that the nature of engagements also varied greatly throughout the project. For instance, engagements related to the design of the tool were more significant during specific sessions (CoD5, CoD7, and CoD8), while they were noticeably less important during other sessions (CoD4, AC2, CoD6, and AC3). A significant majority of the engagements related to the situation involved interaction with others, which is inherent to co-design, as interaction with others is a fundamental characteristic of the process. Figures2  3 suggest that the nature of engagement varied depending on participants’ categories and sessions.

### Example of Data Per Session

Below are examples of empirical data from each session (see Table 2), including session objectives and the methods and materials. The verbatim excerpts are enclosed in quotation marks.

**Table 2.** Session’s location and participants.

Session	Objective	Methods and materials	Data examples
AC^a^1	Validate the design decisions made during the first 2 co-design sessions regarding user needs and address conflicting results.	Plenary discussion with a Microsoft PowerPoint presentation reporting the results of previous co-design sessions and addressing questions about conflicting results.	Situation: Understand the process previously used to compile the list of needs (HSSP^b^ 11‐5)*.* Design of the tool: Determine what will work from the start and understand what caregivers are likely to use. (HSSP 11‐5).
CoD3^c^	Identify user needs already addressed by other tools and determine the functionalities and content requirements related to those needs.	Comparison of existing cyberhealth tools for caregivers (2 websites, 1 app, and 1 video) conducted through small group workshops using a speed dating approach.	Professional needs: Discover tools that can be suggested to the clientele (CW^d^ 3‐3). Design of the tool: Avoid “losing” the caregiver through a complex and challenging resource search process (CW 3‐3).
CoD4	Identify functional and content requirements for needs not addressed by existing tools.	Plenary brainstorming session (1.5 h) followed by small group workshops (1 h) using paper and pens.	Professional needs: Ensure that everyone understands that for CW, the client is not the caregiver but the person receiving assistance (CW 4‐2). Ensure that the health services network can meet the demands of caregivers generated by the tool. “There are two problems. ONE, sometimes you’re entitled to it, but you refuse the service, and TWO, there is a lack of time and resources to provide everything. If people become aware of the services, they will request them, and it will cost too much.” (CW 4‐2)*.* Situation: Redirect the discussion to the intended topic, as participants only had 3 hours, and the conversation had deviated from addressing the primary question (CW 4‐2).
CoD5	Design the information architecture.	Paper prototyping conducted in small group workshops using cardboard, pens, and small pieces of paper to represent options for functionalities and content requirements.	Design of the tool: Avoid information overload by preventing it from becoming a “monster” and ensuring it remains “simple but understandable.” (Caregiver 5‐8). Situation: Assist the caregiver in responding to the facilitating researcher who asked about a filter that could limit search results for resources in their region. He knows that older adults often do not understand computer logic; to them, a filter is akin to a coffee filter (CW 5‐1).
AC2	Decisions on conflicting requirements.	Plenary discussion (3 h) involving both in-person and remote participants, using 2 documents: a 13-page table of results from all previous sessions and a 16-page summary table of requirements to meet the needs (results from the previous session).	Personal needs: Understand the research project and provide relevant interventions, as she lacked sufficient information before the meeting to fully grasp the project (Caregiver 11‐11). Situation: Have a positive experience of participation. "I didn’t fully understand my discomfort during the meeting, but I thought about it, and I realized I had become so anxious because I didn’t want to mess anything up. I reflected a lot on whether I wanted to continue participating in the research because, for sure, with the way I had experienced it, I didn’t want to anymore..." (Caregiver 11‐11).
CoD6	Information design.	Plenary presentation and discussion (1 h), followed by small group brainstorming workshops (1 h and 45 min) using activity sheets for each group.	Personal needs: Be prepared to achieve the objectives. She needed to make a choice but was unable to prepare because she had not received any information about the planned activities (HSSP 6‐6). Situation: Have sufficient time to achieve the objective. "At that moment, I knew we wouldn’t finish our mandate. Instead of pushing forward and trying to say... at that point, I decided to disengage” (HSSP 6‐6).
CoD7	Validation of the information architecture and interaction design.	Small group brainstorming workshops using activity sheets for each group and an interactive low-fidelity prototype (version 1).	Situation: Ensure that a common goal is pursued. "I felt that she [a CW] was attacking your project... [...] Meaning to say: 'You don’t need to do that, we’re already doing it!'" (Caregiver 7‐7). Design of the tool: Feel welcomed and guided by the homepage, “But caregivers need this... They’re tired, they don’t know where to go, they don’t know what to do” (Caregiver 7‐7).
CoD8	Comparison of homepage proposals, examination of the search results page, and validation of questions designed to help identify needs.	Small group brainstorming workshops followed by a usability discussion using an interactive low-fidelity prototype (version 2).	Design of the tool: Ensure that visually, it immediately captures attention (Caregiver 8‐9). Avoid returning search results with no findings (Caregiver 8‐9).
AC3	Decisions on conflicting results. Obtain feedback on the latest version of the prototype before website programming.	Presentation of a medium-fidelity prototype (version 3) in small groups (30 min), followed by a plenary discussion (2 h).	Professional needs: Ensure that the language and terms used are appropriate. “I always have a concern about the language and terms that we use.” (CW 11‐6). Situation: Take a stance without fear of judgment. "We’re taking a stance on this, and that’s okay. We don’t have to be judged for it. [...] Can we have our approaches without getting jumped on?” (CW 11‐6)

a AC: advisory committee meeting.

b HSSP: health and social service professionals.

c CoD: co-design session.

d CW: community worker.

## Discussion

### Principal Findings

Our objective was to obtain an intrinsic description of engagement, encompassing what motivates and concerns actors in a co-design situation, from their perspective. The chosen methodology, grounded in a pragmatic paradigm and informed by the course-of-action framework, allowed us to foreground participants’ lived experiences and situated knowledge. This approach privileged perspectives that are often underrepresented in more top-down design processes. By adopting a co-design approach supported by self-confrontation interviews, we were able to access not only what participants said during the sessions but also how they made sense of their own engagement. These perspectives influenced both the iterative refinement of the design and our interpretation of the engagement dynamics.

The results might not appear surprising, yet it seems we are still encountering the same challenges in co-design projects. The contribution of this paper is to emphasize these results with a detailed description of the methodology and empirical data evaluating participant engagement, as recommended by Manafo et al [29] and Slattery et al [8]. This includes describing what was significant in participants’ overall experience, from their perspective. Our analysis highlights that the nature of participants’ engagement during the sessions went far beyond the design of the digital health tool for caregivers, even though that was the reason they were invited to participate. This aspect has not been discussed in other studies.

### Comparison With Previous Work

Among the commonly discussed findings, our study revealed that actors need preparation (personal need) to understand their role, which enhances their sense of relevance and their ability to contribute meaningfully [7,8,27,29,31]. Service providers were also engaged in increasing their knowledge (professional needs) [8,13]. A unique finding was the engagement of community workers, who sought to ensure their distinct concerns—differing from those of HSSPs—were understood. This is particularly relevant in Quebec, where community organizations operate under a model of entrepreneurial governance, with private foundations influencing their mission and work [48]. These engagements related to professional needs eventually led to engagements related to the situation (interaction with others).

Several engagements related to the situation mirrored those identified by Zowghi et al [7]. Unlike common issues of power distribution reported in other studies, our research did not encounter such conflicts between researchers and participants. However, we observed tensions between HSSPs and community workers, particularly during the final session (AC3), highlighting the need for a positive environment, mutual respect [29], and a shared vision [8]. Participants emphasized the importance of having sufficient time to achieve objectives [8,27].

### The Nature of Activity as an Affordance of Action and Engagement

The concept of affordance [49] highlights perceptual cues in the environment that suggest specific interactions. From this perspective, we propose that the activities offered to participants shaped the nature of their engagement in this project. Plenary discussions generally fostered engagements related to personal and professional needs, as well as situational aspects. In contrast, design-centric activities primarily directed engagements toward the tool’s design.

**Figure 4.**
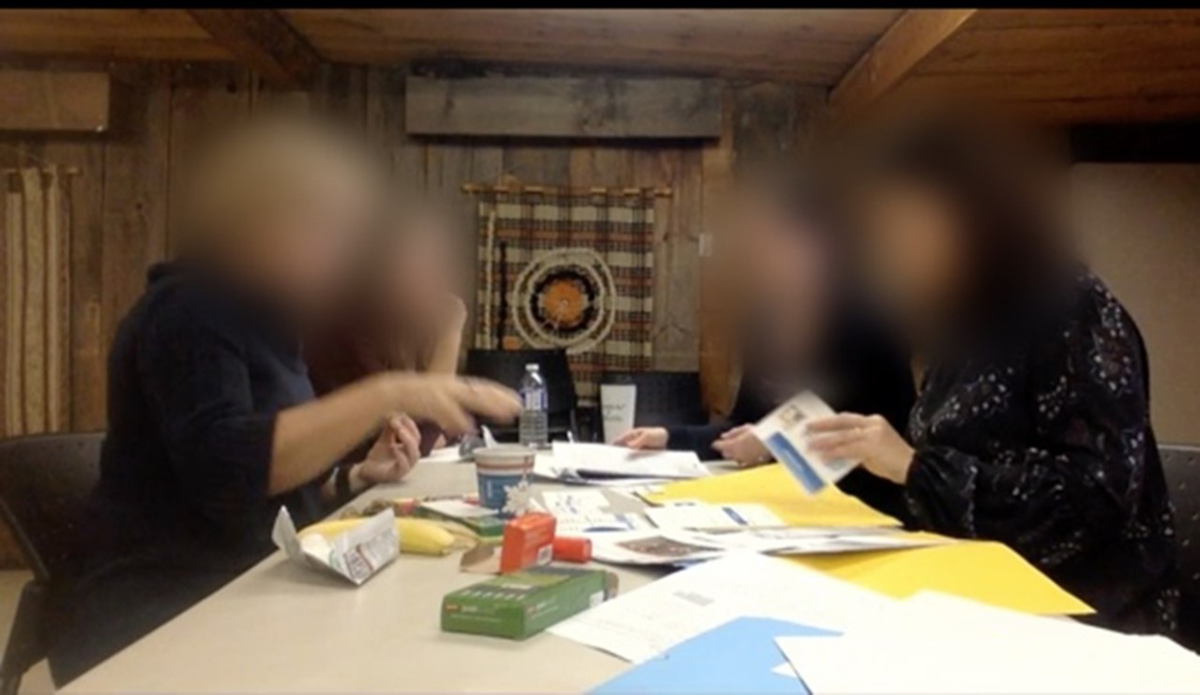
Paper prototyping activity.

Participants in our study who were more engaged in the tool’s design participated in typical design activities, such as paper prototyping (see Figure 4), mockups, clickable prototypes, and usability testing. These activities, which enhance creativity and direct involvement, acted as affordances by guiding appropriate actions, driving engagement, and directing participation (acting, thinking, or speaking) toward the tool’s design. Effective facilitation of co-design sessions should emphasize design practices [50,51], aligning with best practices in this field, as reviewed by Mosely et al [52]. This approach supports the “designing with” philosophy rather than “designing for,” as suggested by Howard and Somerville [25].

### Establishing an Engagement-Focused Approach

Grasping the essence of engagement is key to understanding participation in co-design, a core element of participatory research. However, the question arises: participation in what, exactly? From a researcher’s viewpoint, participation implies committed involvement in co-designing a product or service. Yet, our findings suggest that participants perceive participation differently, emphasizing the diversity of engagement. This includes concerns, situational considerations, and the mobilization of their participation. In terms of research ethics and co-design, we should consider engagements that prioritize participants’ needs and the situation. Adopting a compassionate stance to meet their expectations allows them to derive benefits from engagements that appear more significant to them. Based on our results, we recommend that researchers and designers using a co-design approach establish conditions that respect the diverse nature of participant engagements. To meet expectations tied to participants’ personal needs and enable them to benefit from their involvement [22], we propose exploring the following avenues (see Textbox 3).

**Figure 5.**
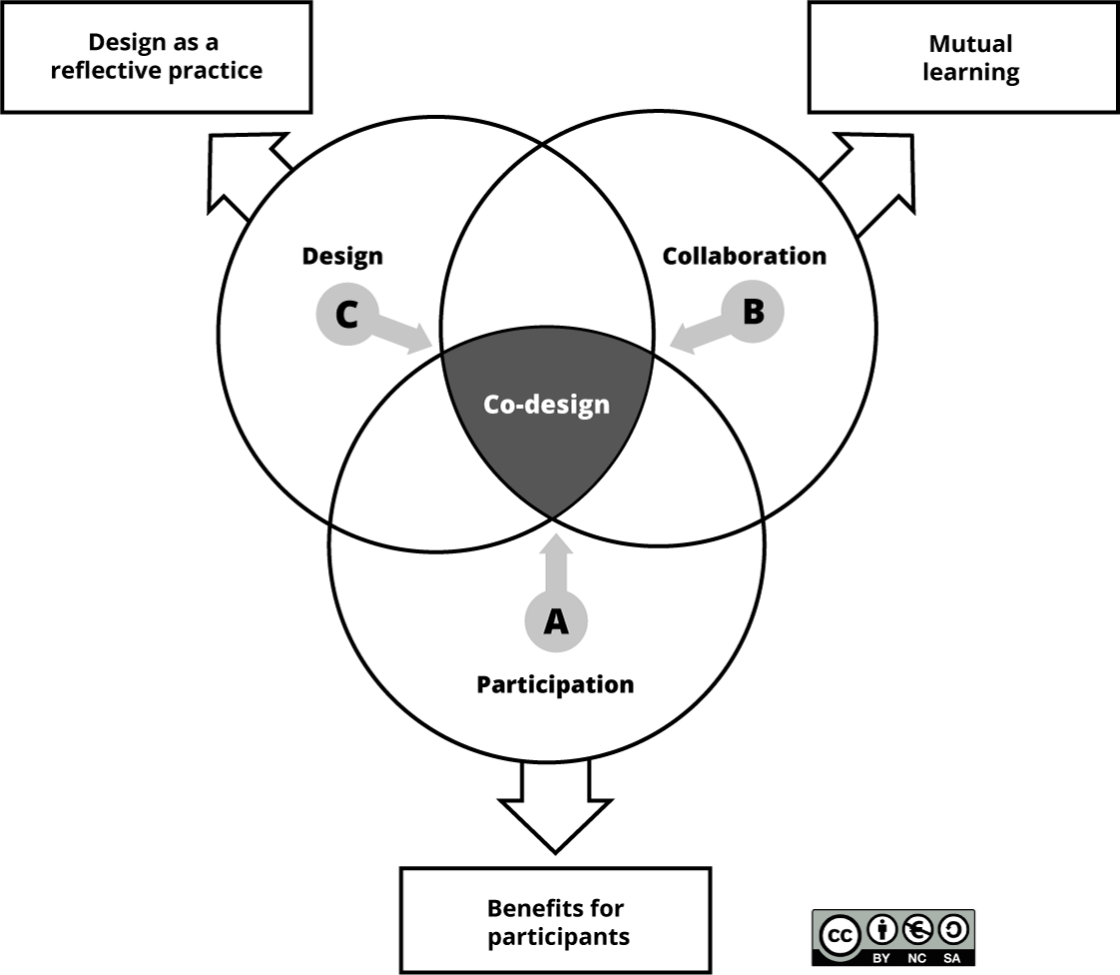
Balancing the tensions between the dimensions of co-design.

As mentioned earlier, co-design, for us, lies at the intersection of 3 dimensions: design, collaboration, and participation. These dimensions are closely related, resembling a Venn diagram. The tensions between these dimensions will impact the center (co-design). The suggested affordances (A, B, and C) could help balance these tensions (see Figure 5).

Textbox 3. **Affordances that enable participants to benefit from their participation.**

1. Consider participants’ personal and professional engagements:
  - Recognize that participants pursue personal and professional goals that may extend beyond the intended design objectives for which they were invited to contribute.
  - Allow participants to express their personal and professional engagements (or expectations) by dedicating a specific period during sessions.
  - Endeavor to address expectations related to personal and professional engagements to honor the goals they pursue through their participation.
2. Address expectations related to the situation:
  - Ensure a shared objective from the outset by identifying and including all interest holders from the beginning.
  - Embrace a flexible approach to achieve objectives by allocating sufficient time and offering a satisfactory experience to participants.
  - Enable participants to comprehend the discussed elements by ensuring they have the necessary knowledge, such as by sending them a preparatory sheet before the session, for example, as the flipped classroom approach in education.
  - Ensure the maintenance of a positive environment characterized by mutual respect during all sessions.
3. Configure activities as affordances that suggest appropriate actions:
  - Prioritize design activities that guide participants’ actions toward tool design.
  - Prompt participants to engage in action quickly during sessions (active mode) and avoid prolonged presentations (passive mode).
  - Adopt best practices in design facilitation.

### Limitations

Participants varied in each co-design session. Analyzing the engagement of individuals who participated throughout the entire process using the course-of-action framework might yield different results. Comparing our findings with those of continuous participation could enrich the discussion on engagement by exploring the similarities and differences arising from the methodological choice of isolated versus continuous or multiple participation.

### Conclusions

In this study, we explored the concept of engagement among 3 categories of potential users—caregivers, HSSPs, and organizations—who participated in a cyberhealth co-design process. We used a robust methodological approach based on the course-of-action framework to document participant engagements, addressing a gap in the scientific literature [8]. Our findings reveal that engagement extends beyond the immediate goals of tool design, encompassing broader concerns and motivations shaped by individual needs and the dynamics of interaction. In my professional co-design practice, I have integrated these recommendations by adopting a compassionate stance that prioritizes participants’ perspectives and by configuring activities as affordances to support meaningful engagement. This approach has consistently led to more inclusive and impactful co-design processes, as evidenced by the quality of participation and the relevance of the outcomes generated. Our analysis highlights the importance of balancing design, collaboration, and participation to address the inherent tensions in co-design projects. Ultimately, this study underscores the value of tailoring co-design practices to respect and leverage the unique contributions of all participants, thereby advancing both the process and outcomes of collaborative innovation.

## Abbreviations

AC: advisory committee session
CoD: co-design session
HSSP: health and social services professional
